# Retrospective Analysis of the Clinical Findings of 114 Horses with Eosinophilic Keratoconjunctivitis

**DOI:** 10.3390/ani16192982

**Published:** 2026-09-22

**Authors:** József Tóth, Lieke Buijs, Rike Lorenz, Zoltan Bakos

**Affiliations:** 1 Tierärztliches Kompetenzzentrum Karthaus GmbH, 48249 Dülmen, Germany; toth.jozsef@univet.hu (J.T.); dr.buijs@tierklinik-karthaus.de (L.B.); 2 Department and Clinic of Equine Medicine, University of Veterinary Medicine Budapest, Dora Major, H-2225 Üllő, Hungary; rike-lorenz@web.de

**Keywords:** equine, keratitis, conjunctivitis, hypersensitivity, eosinophil, retrospective case series

## Abstract

Eosinophilic keratoconjunctivitis (EKC) is an inflammatory eye disease of horses with an unknown cause and suspected immune-mediated pathogenesis. This single-center retrospective case series included 114 horses (163 affected eyes) examined at a German equine referral hospital from January 2012 to December 2020. Of these horses, 71 (62.3%) were Warmbloods, 77 (67.5%) were presented from July to September, and 49 (43.0%) had bilateral disease. The cornea was involved in 139 of 163 eyes (85.3%). During the study period, 114 EKC cases occurred among 5742 ophthalmology cases (2.0%), with an increasing overall trend in hospital-based relative frequency (*p* < 0.001). All horses received medical treatment, and 90 (78.9%) also underwent surgery. Follow-up was available for 98 of 114 horses (86.0%): 78 (79.6%) recovered, 12 (12.2%) had persistent disease, and 8 (8.2%) underwent enucleation. Among recovered horses, median recovery time was 30 days. Exploratory analyses associated surgery with longer recovery (*p* < 0.001) and corneal lesion morphology with adverse outcome (*p* = 0.020), but treatment was clinician-selected. These findings describe EKC in one referral-hospital population but do not establish population incidence, seasonal risk, breed predisposition, or comparative treatment efficacy.

## Simple Summary

Eosinophilic keratoconjunctivitis is an eye disease in horses that can affect the clear surface of the eye (cornea), the surrounding tissue (conjunctiva), and the third eyelid. Its cause is unknown, although an abnormal immune response is suspected. We reviewed records from 114 affected horses treated at a German referral hospital between 2012 and 2020. Most were presented from July to September, and almost half had disease in both eyes. All horses received medication, and most also underwent an eye procedure or surgery. Follow-up was available for 98 horses: 78 recovered, 12 had continuing disease, and 8 required removal of the affected eye. Of the horses that recovered, half recovered within about one month, although recovery times varied widely. These findings may help veterinarians recognize the disease and explain its possible course to owners. Because the study involved one referral hospital, it cannot show how common the disease is in the wider horse population, whether it is truly seasonal, or which treatment works best.

## 1. Introduction

Eosinophilic keratoconjunctivitis (EKC) is a relatively rare ocular disease occurring in humans, cats, rabbits, dogs, donkeys, and horses [1,2,3,4,5,6,7,8]. The first equine case was reported in the USA in 1994 [9]. In horses, the initiating etiology remains unknown [10]. The disease may involve the cornea, conjunctiva, and third eyelid [11]. Nevertheless, the prominent eosinophilic infiltrate and proposed type I and/or type IV hypersensitivity responses support a suspected immune-mediated mechanism, potentially triggered by an unidentified environmental or parasitic antigen [8,12]. Activated eosinophils release cytotoxic proteins and matrix metalloproteinase-9, which may damage corneal and conjunctival epithelial cells [12].

Clinical expression is variable and may be unilateral or bilateral. Common findings include ocular discomfort, blepharospasm, epiphora, conjunctival hyperemia and chemosis, corneal edema, and white-to-yellow caseous or mucopurulent material [11,13,14]. A tentative diagnosis can be based on the ocular appearance, but cytologic or histopathologic demonstration of eosinophils in corneal, conjunctival, or third-eyelid tissue is required for confirmation [13,15].

Reported management combines topical and systemic anti-inflammatory treatment with antimicrobial therapy when secondary infection is suspected or confirmed [11,12,16]. Nasolacrimal duct lavage may be used when thick exudate accumulates [17]. Superficial keratectomy, diamond burr debridement, and diode laser treatment have also been used, particularly for persistent plaques, ulceration, or inadequate response to medical management [8,10,12,18]. Because controlled comparative evidence is limited, treatment selection is largely guided by lesion features, severity, clinician judgment, and response to initial therapy.

Some authors have linked broader increases in immune-mediated disease to environmental change [19], and long-term climatic changes have been documented globally and in North Rhine-Westphalia [20,21,22]. However, meteorological exposures were not linked to individual cases in the present study, and any climatic contribution to EKC remains an untested hypothesis.

Previous equine reports range from small case descriptions to retrospective series of 27–29 horses and describe variable clinical forms, a frequent concentration of presentations in warmer months, prolonged recovery in some cases, and occasional recurrence [8,14,23,24,25]. Larger clinical cohorts are needed to characterize the spectrum of presentation, management, and documented outcomes more precisely. This retrospective case series aimed to describe the signalment, temporal distribution of presentations, ocular involvement, lesion morphology, microbiological findings, management, and documented outcomes of horses with EKC examined at a single German equine referral hospital.

## 2. Materials and Methods

### 2.1. Study Design and Case Selection

This retrospective single-center case series included horses examined and treated at the Tierärztliche Klinik für Pferde Domäne Karthaus in Dülmen, North Rhine-Westphalia, Germany, between 1 January 2012 and 31 December 2020. The clinic is a regional equine referral hospital; therefore, the cohort represents the hospital’s regional referral population, not the general German equine population.

Eligible cases were identified from electronic medical records (easyVet, VetZ GMBH, Isernhagen, Germany) using the diagnosis field. This search provided 114 horses for analysis. After conducting the search, we verified the clinical data in the electronic records against the diagnosis. Inclusion required clinical findings compatible with EKC and histopathologic confirmation of eosinophilic inflammation in an ocular sample. Each horse had a complete ophthalmic examination report upon arrival, along with details of the treatment modalities received during hospitalization. Based on the available historical data at admission, these horses had no previous or concurrent ocular disease.

### 2.2. Ophthalmic Examination and Diagnostic Sampling

All horses underwent a detailed ophthalmic examination by a professor of veterinary ophthalmology (JT), including slit-lamp biomicroscopy and ocular ultrasonography. Corneal, conjunctival, and/or third-eyelid samples were submitted for histopathologic examination according to lesion location. In ulcerative eyes, the eroded and necrotic tissue surrounding the ulcer was excised. In pseudomembranous cases, the sample was removed by scraping the pseudomembrane using the blunt end of the scalpel. In granulomatous eyes as well as in cases with conjunctivitis or third eyelid involvement, the sample was excised using iris scissors. Histopathological results were available approximately 24 h later after accelerated histological processing.

Microbiological testing was performed in 47/114 horses (41.2%). The attending clinician decided on sampling based on the clinical signs. Bacterial culture was done in all samples. Fungal culture was requested when histopathology confirmed fungal elements in the samples. Culture positivity was calculated using the number tested as the denominator.

### 2.3. Variables, Statistical Units, and Outcome Definitions

Extracted horse-level variables were age, breed, sex, year and month of presentation, laterality, treatment category, follow-up availability, recovery time, persistent/chronic disease, enucleation, and documented recurrence. The reported month and year represent presentation to the study hospital rather than verified disease onset. Eye-level variables were affected ocular structures, lesion morphology, treatment category, outcome, and recovery time. For bilateral horses, a horse was classified as recovered only when both eyes had documented recovery. A horse with an enucleated eye was classified in the enucleation group. Horses were considered to have chronic disease when their clinical signs persisted for a long time, and their owners were unable to continue their hospital treatment due to financial constraints; hence, they were transported home to continue their medical treatment there. If recovery of one or both eyes was unknown, the horse-level outcome was classified as unknown unless an adverse outcome was documented. Annual ambulatory and hospitalized ophthalmology counts were available for 2012–2020 and were summed for the annual denominator. Monthly ophthalmology caseload denominators were unavailable. Analyses and denominators were reported at the horse or eye level as appropriate.

Recovery was defined as complete corneal re-epithelialization, including negative fluorescein staining, along with resolution of eosinophilic lesions and clinically significant inflammation. Recovery time was measured from the first examination at the study hospital to the examination documenting recovery. For bilaterally affected horses, horse-level recovery time was defined as the interval until both eyes met the recovery criteria. For statistical analysis, adverse outcome was defined as persistent/chronic disease or enucleation. Horses or eyes with an unknown outcome were excluded from the corresponding outcome analysis.

Follow-up was considered available when a re-examination, referring veterinarian report, or owner communication documented outcome. Recurrence was defined as a subsequent episode of clinically diagnosed EKC recorded at the study hospital after documented recovery. Because horses were not contacted systematically and treatment elsewhere could not be captured, recurrence was described as documented recurrence rather than estimated population recurrence.

### 2.4. Treatment Protocol and Classification

Treatment information was extracted from individual medical records. For descriptive reporting and exploratory treatment–outcome analyses, horses were classified at the horse level as receiving either medical treatment alone or medical treatment combined with at least one surgical procedure. Horses undergoing any surgical procedure during hospitalization were assigned to the medical-plus-surgery group. Because this was a retrospective study, treatment was selected by the attending clinician according to clinical judgment and was not randomly assigned according to predefined study criteria.

The medical treatment protocol comprised oral flunixin meglumine (Flunidol 5% gel, CP-Pharma, Burgdorf, Germany) at 1.1 mg/kg twice daily for 3 days, followed by 0.55 mg/kg for 3–5 days and 0.275 mg/kg for a further 3–5 days. Oxytetracycline ophthalmic ointment (Oxytetracyclin-Augensalbe 10 mg/g, Jenapharm, Jena, Germany) was initially applied three times daily for at least 5 days. After the acute inflammation subsided, this treatment was gradually replaced with an ointment containing oxytetracycline and prednisolone (Oxytetracyclin-Prednisolon-Augensalbe 5 mg/g + 2.5 mg/g, Jenapharm, Jena, Germany), starting once daily and increasing to three times daily under close clinical monitoring.

When secondary anterior uveitis was present, 2–3 mg atropine sulfate (Atropinsulfat 100 mg injection, Dr. F. Köhler Chemie, Bensheim, Germany) was administered subconjunctivally. After the acute phase, subconjunctival injections containing 0.6–1.2 mg dexamethasone sodium phosphate (Hexadreson 2 mg/mL injection, MSD Animal Health, Unterschleißheim, Germany) and 10–15 mg gentamicin (Genta 100 mg/mL injection, CP-Pharma, Burgdorf, Germany) were administered at 5-day intervals under close clinical monitoring. Surgical procedures were further classified as superficial keratectomy, diode laser surgery, diamond burr debridement, or combined diamond burr debridement and diode laser surgery.

### 2.5. Statistical Analysis

Analyses were performed in Microsoft Excel for Microsoft 365 MSO (Version 2608 Build 16.0.20326.20072) and IBM SPSS Statistics version 27.0.1. Categorical variables were summarized as counts and percentages using the relevant horse- or eye-level denominator. Continuous variables were assessed for normality with the Shapiro–Wilk test. Normally distributed data were summarized as mean ± standard deviation (SD), and non-normally distributed data as median, interquartile range (IQR), and range. A one-way analysis of variance was used to compare age among mares, geldings, and stallions, and a Kruskal–Wallis test compared recovery time among sex categories. Spearman rank correlation assessed the association between age and recovery time among recovered horses.

The horse was the statistical unit for treatment and age analyses. Among the 98 horses with documented follow-up, Fisher’s exact test compared treatment category with the binary outcome of recovery versus adverse outcome (persistent/chronic disease or enucleation). Among the 78 recovered horses, the Mann–Whitney U test compared recovery time between medical-only and surgical treatment groups. The eye was the unit for exploratory lesion-form analyses, which included the 139 eyes with corneal involvement and classified lesions as ulcerative, pseudomembranous, granulomatous, or mixed. Unknown eye outcomes were excluded from outcome analysis. Logistic regression assessed lesion form in relation to adverse outcome among 125 eyes with known outcomes. Recovery duration was log-transformed and analyzed using linear regression among 100 recovered corneal eyes. Overall associations were evaluated with Wald tests. Recovery time analyses excluded chronic, enucleated, and unknown cases. A significance level of *p* < 0.05 was assumed for these tests.

Annual EKC relative frequency for 2012–2020 was calculated as the number of EKC presentations divided by the total ophthalmology caseload for each year. Wilson 95% confidence intervals were calculated for the annual proportions, and an ordered temporal trend was assessed using the Chi-square test for trend. All 114 cohort horses were included in this analysis.

## 3. Results

### 3.1. Study Population and Temporal Distribution of Presentations

The study included 114 horses with 163 affected eyes. Cohort characteristics, ocular involvement, management, and documented outcomes are summarized in Table 1. Age ranged from 1 to 22 years and was normally distributed (Shapiro–Wilk *p* = 0.062), with a mean ± standard deviation of 11.6 ± 5.1 years. The largest age category was 11–15 years (42/114; 36.8%) (Figure 1). Age differed among sex categories (one-way analysis of variance, *p* < 0.001); stallions were younger (3.33 ± 2.83 years) than mares (11.9 ± 5.3 years) and geldings (12.6 ± 4.1 years). Eight of 15 horses aged 0–5 years (53.3%) were stallions.

The cohort comprised 71 Warmbloods (62.3%), 24 ponies (21.1%), 5 Quarter Horses (4.4%), 4 Thoroughbreds (3.5%), and 2 horses each (1.8%) of the Andalusian, Arabian, Friesian, Paint Horse, and Standardbred breeds. There were 52 mares (45.6%), 53 geldings (46.5%), and 9 stallions (7.9%). No external reference population was available for breed or sex comparisons.

During 2012–2020, 114 EKC cases were identified among 5742 ophthalmology cases (overall relative frequency, 2.0%). Annual relative frequency increased from 1/524 ophthalmology cases (0.2%) in 2012 to 26/685 (3.8%) in 2018, and was 20/754 (2.7%) in 2019 and 20/765 (2.6%) in 2020 (Figure 2). The Chi-square test indicated an increasing ordered trend across 2012–2020 (*p* < 0.001). These values describe relative frequency within the study hospital’s ophthalmology caseload and do not estimate population incidence.

Presentation month was concentrated from July through September (77/114; 67.5%), with the largest monthly count in August (29/114; 25.4%) (Figure 3). The month represents presentation to the study hospital, not necessarily disease onset. Without monthly reference population denominators, this distribution does not establish increased seasonal risk.

### 3.2. Laterality, Ocular Structures, and Lesion Morphology

Disease was unilateral in 65/114 horses (57.0%) and bilateral in 49/114 (43.0%), yielding 163 affected eyes. Among unilateral cases, the left eye was affected in 32/114 horses (28.1%) and the right eye in 33/114 (28.9%). The cornea alone was affected in 84/163 eyes (51.5%), the cornea and conjunctiva in 48/163 (29.5%), the cornea and third eyelid in 7/163 (4.3%), and the conjunctiva alone in 24/163 (14.7%).

Among the 139 eyes with corneal involvement, lesions were purely ulcerative in 54/163 eyes (33.1%), pseudomembranous/diphtheroid in 29/163 (17.8%), and granulomatous in 23/163 (14.1%). Mixed ulcerative-pseudomembranous, ulcerative-granulomatous, and granulomatous-pseudomembranous lesions occurred in 28/163 (17.2%), 3/163 (1.8%), and 2/163 eyes (1.2%), respectively. The remaining 24/163 eyes (14.7%) had eosinophilic conjunctivitis without corneal involvement. Representative ulcerative, pseudomembranous, and granulomatous lesions are shown in Figure 4, Figure 5 and Figure 6.

### 3.3. Microbiological Findings

Among 47 horses in which microbiological testing was performed, 6 (12.8%) had positive bacterial cultures, and 5 (10.6%) had positive fungal cultures. Bacterial isolates were Staphylococcus aureus (four samples) and Staphylococcus haemolyticus (two samples); fungal isolates were Aspergillus species (five samples).

### 3.4. Management

All 114 horses received the medical protocol described above. Twenty-four horses (21.1%) received medical treatment alone. An additional surgical procedure was performed in 90 horses (78.9%): superficial keratectomy in 71 (62.3%), diode laser surgery in 12 (10.5%), diamond burr debridement in 6 (5.3%), and combined diamond burr debridement and laser surgery in 1 (0.9%). These frequencies describe clinician-selected management and were not used to infer comparative efficacy.

### 3.5. Follow-Up and Outcomes

Follow-up information was available for 98/114 horses (86.0%) and unavailable for 16/114 (14.0%). Of the 98 horses with follow-up, 78 (79.6%) had documented recovery, 12 (12.2%) had persistent/chronic disease without complete recovery, and 8 (8.2%) underwent enucleation because of persistent severe clinical disease (Figure 7).

Among the 78 horses with documented recovery, recovery time was non-normally distributed (Shapiro–Wilk *p* < 0.001), with a median of 30 days (IQR, 21–60 days; range, 5–330 days). Age was not correlated with recovery time (Spearman rho = 0.004, *p* = 0.969), and recovery time did not differ among sex categories (Kruskal–Wallis H = 0.32, *p* = 0.851). Treatment- and lesion-specific outcomes and recovery times are summarized in Table 2.

In exploratory horse-level analyses, median recovery time was 20.5 days (IQR, 14–30 days) in the medical-only group and 45 days (IQR, 30–60 days) in the surgical group (Mann–Whitney U = 293.5, *p* < 0.001). Among horses with known outcomes, adverse outcomes occurred in 0/22 medically managed horses (0%) and 20/76 horses that underwent surgery (26.3%; Fisher’s exact *p* = 0.005). Because treatment was clinician-selected and likely influenced by disease severity and lesion morphology, these associations do not estimate a causal treatment effect.

In eye-level analyses restricted to corneal lesions, morphology was associated with adverse outcome after accounting for clustering of bilateral eyes within horses (overall Wald *p* = 0.020). Compared with purely ulcerative lesions, adverse outcome odds were higher for pseudomembranous lesions (odds ratio, 7.34; 95% confidence interval, 1.85–29.15) and mixed lesions (odds ratio, 5.04; 95% confidence interval, 1.20–21.10), but not granulomatous lesions (odds ratio, 1.47; 95% confidence interval, 0.23–9.47). Lesion morphology was not associated with recovery duration among the 100 recovered corneal eyes (overall Wald *p* = 0.249). These sparse, unadjusted results are exploratory.

Two of 98 horses with available follow-up (2.0%) had a recurrence documented at the study hospital; one horse had one documented recurrence, and the other had two. Because follow-up was not based on systematic long-term surveillance, this proportion may underestimate recurrence.

## 4. Discussion

This retrospective case series describes 114 horses and 163 eyes with histopathologically confirmed EKC from one German equine referral hospital. The cohort is larger than previously published equine case series of 27–29 horses [8,23,25]. Principal clinical findings included frequent bilateral involvement, heterogeneous corneal and conjunctival lesion morphology, wide variation in time to documented recovery, and the need for enucleation in a minority of horses. These observations characterize the study cohort but should not be interpreted as population-level measures of risk or comparative treatment efficacy.

Age was broadly distributed and the mean age (11.6 years) was similar to values reported previously [8,23,25]. Age was not correlated with recovery duration among recovered horses. Warmbloods comprised 62.3% of this cohort, but no clinic or regional reference population was available. Their frequency therefore cannot demonstrate breed predisposition and may reflect the case mix of the referral hospital. The near-equal numbers of mares (52) and geldings (53) likewise do not establish a sex association. The small number of stallions is plausibly influenced by husbandry and referral patterns rather than disease biology.

Most presentations occurred from July through September, consistent with the warmer-month clustering reported in previous case series [8,23,25]. However, presentation month may differ from true disease onset, and monthly ophthalmology-caseload denominators were unavailable. The present data therefore show a seasonal distribution of presentations, not increased seasonal risk. Annual ophthalmology denominators were available and showed that the relative frequency of EKC within the hospital’s ophthalmology caseload increased across the study period, although it peaked in 2018 and was lower in 2019–2020. This hospital-based trend does not establish population incidence or prove that EKC is an emerging disease. Changes in referral patterns, clinical awareness, diagnostic practice, or case ascertainment could have contributed. Although long-term climatic change has been documented in North Rhine-Westphalia [20,21,22], no meteorological exposure variables were linked to individual cases and no climate-disease association was tested. Any climatic contribution remains a hypothesis for future study.

Bilateral disease occurred in 43.0% of horses, confirming that involvement of both eyes is clinically important, although the proportion was lower than the 62.1% reported by Knickelbein et al. [8]. Corneal involvement predominated, while 14.7% of eyes had conjunctival disease alone. The ulcerative, pseudomembranous/diphtheroid, granulomatous, and mixed patterns provide a practical morphologic description of the spectrum of EKC [10,12]. Pseudomembranous and mixed lesions had higher adverse-outcome odds than purely ulcerative lesions in the exploratory cluster-adjusted analysis, whereas recovery duration did not differ significantly among lesion forms. Sparse adverse outcomes, wide confidence intervals, retrospective classification, and lack of adjustment for severity limit this finding. Because the initiating etiology remains unknown, these forms should be viewed as clinical phenotypes within a suspected immune-mediated disorder rather than proven distinct disease entities.

Positive bacterial or fungal cultures were obtained from 11/47 cultured horses (23.4%), compared with approximately 35% reported in previous studies [11,23,24]. This numerical difference should be interpreted cautiously because only a subset of horses underwent microbiological examination, and testing was not standardized. These findings support the occurrence of secondary microbial involvement in some cases. Culture results should guide antimicrobial decisions where possible, but these retrospective data cannot determine whether prophylactic antibacterial or antifungal treatment is beneficial.

All horses received medical therapy, and 78.9% underwent an additional surgical procedure, most commonly superficial keratectomy. The rationale for corticosteroid therapy is suppression of ocular inflammatory mediators [26], whereas equine reviews describe medical and surgical management of eosinophilic keratitis [27,28]. Surgically treated horses had longer recovery times among recovered cases and a higher proportion of adverse outcomes among horses with follow-up. This pattern should not be interpreted as harm from surgery. Treatment was selected by the attending clinician rather than assigned prospectively, and lesion severity, extent, morphology, duration, secondary infection, and early response could have influenced both treatment selection and outcome. Residual confounding by indication is therefore likely, and the data cannot establish superiority or comparative effectiveness of either modality.

Among horses with documented recovery, median recovery time was 30 days but ranged from 5 to 330 days, consistent with the wide ranges reported in earlier studies [8,25]. Persistent/chronic disease was documented in 12 horses and enucleation in 8, underscoring that clinical outcome is not uniformly benign. Only two horses returned to the study hospital with documented recurrence. This should not be interpreted as a true recurrence rate because horses were not monitored systematically, may have been treated elsewhere, or may not have returned after discharge.

This study has several limitations. Its retrospective, single-center design resulted in non-standardized recording, heterogeneous diagnostic and therapeutic pathways, and missing or incomplete follow-up. Requiring histopathologic confirmation may have preferentially selected severe, atypical, surgically treated, or diagnostically challenging cases and may underrepresent milder cases managed without tissue sampling. Monthly, regional, and national ophthalmology denominators were unavailable, precluding inference about breed or sex predisposition, seasonal risk, or population incidence. Treatment was not randomized, and the exploratory treatment analyses were unadjusted for severity, lesion extent, disease duration, or prior response. Although cluster-robust analyses accounted for non-independence of bilateral eyes, lesion-specific adverse outcomes were sparse and estimates were imprecise. Recovery-time analyses conditioned on documented recovery, and recurrence surveillance was incomplete. Finally, environmental and climatic variables were not analyzed, so no causal relationship with disease occurrence can be inferred.

## 5. Conclusions

Within this single-center retrospective case series of histopathologically confirmed EKC, clinical presentation was heterogeneous, bilateral disease was common, and recovery time varied widely. From 2012 to 2020, the annual relative frequency of EKC within the hospital’s ophthalmology caseload increased, but this does not establish increasing population incidence. The distributions of breed, sex, and presentation month remain descriptive of this referral cohort. Exploratory associations between clinician-selected surgery, lesion morphology, recovery time, and adverse outcome require prospective confirmation and should not be interpreted as causal treatment effects. Multicenter studies with defined reference populations, standardized outcome criteria, systematic follow-up, and adjustment for disease severity are needed.

## Figures and Tables

**Figure 1:**
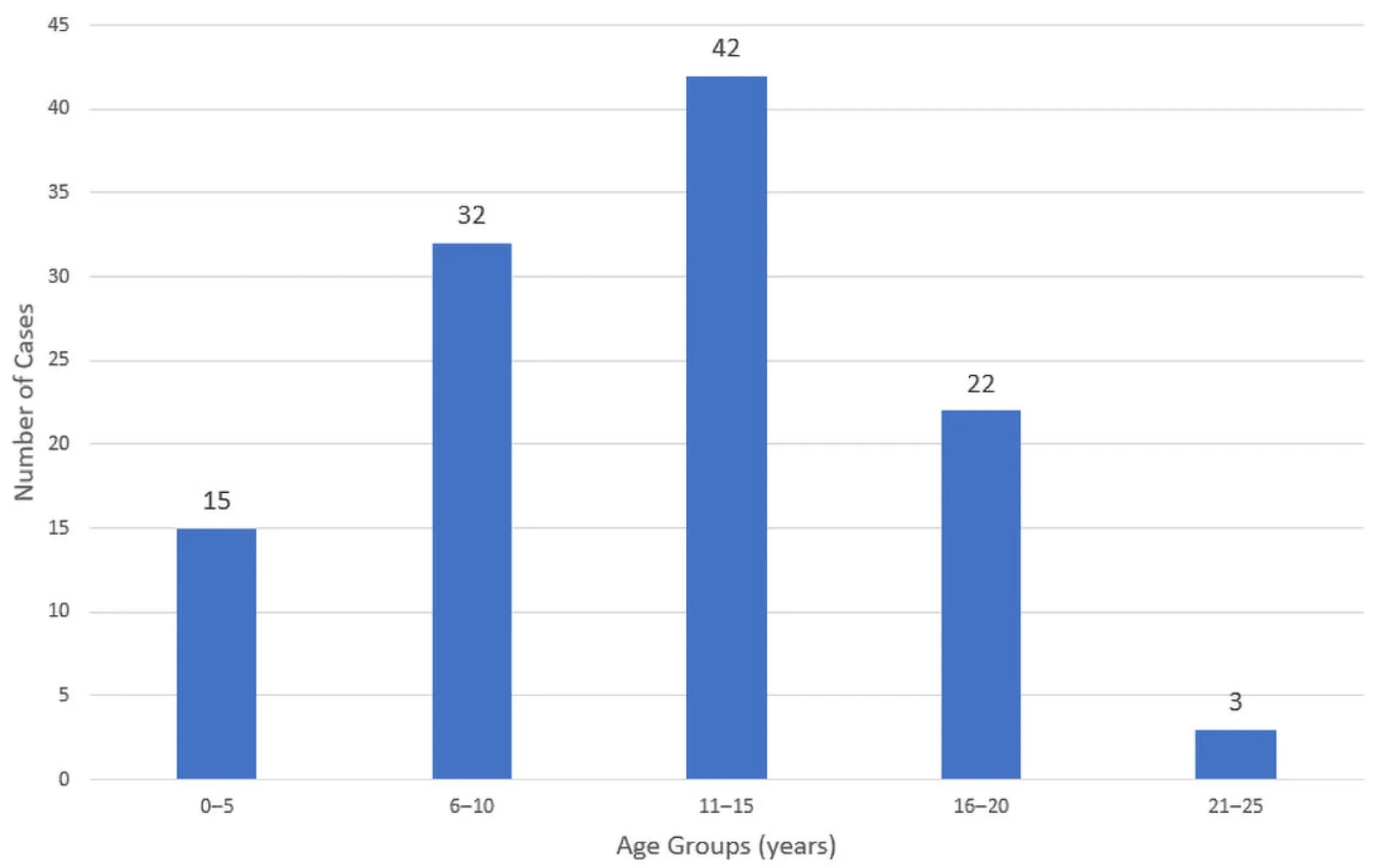
Age distribution of horses with eosinophilic keratoconjunctivitis (n = 114): 0–5 years, 15/114 (13.2%); 6–10 years, 32/114 (28.1%); 11–15 years, 42/114 (36.8%); 16–20 years, 22/114 (19.3%); and 21–25 years, 3/114 (2.6%).

**Figure 2:**
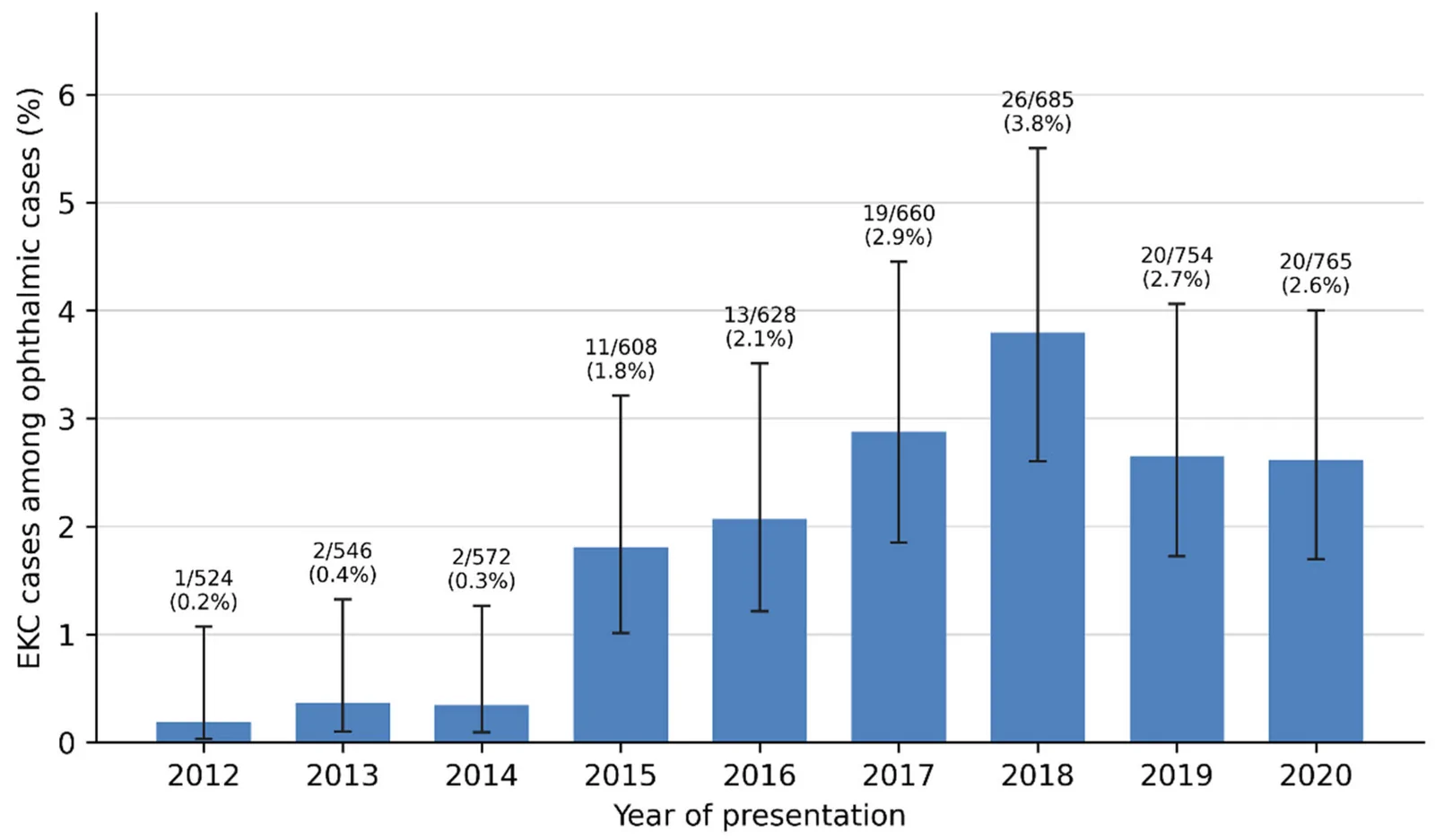
Annual relative frequency of equine eosinophilic keratoconjunctivitis (EKC) among all ophthalmology cases at the study hospital. Denominators combine ambulatory and hospitalized ophthalmology cases. Error bars show Wilson 95% confidence intervals.

**Figure 3:**
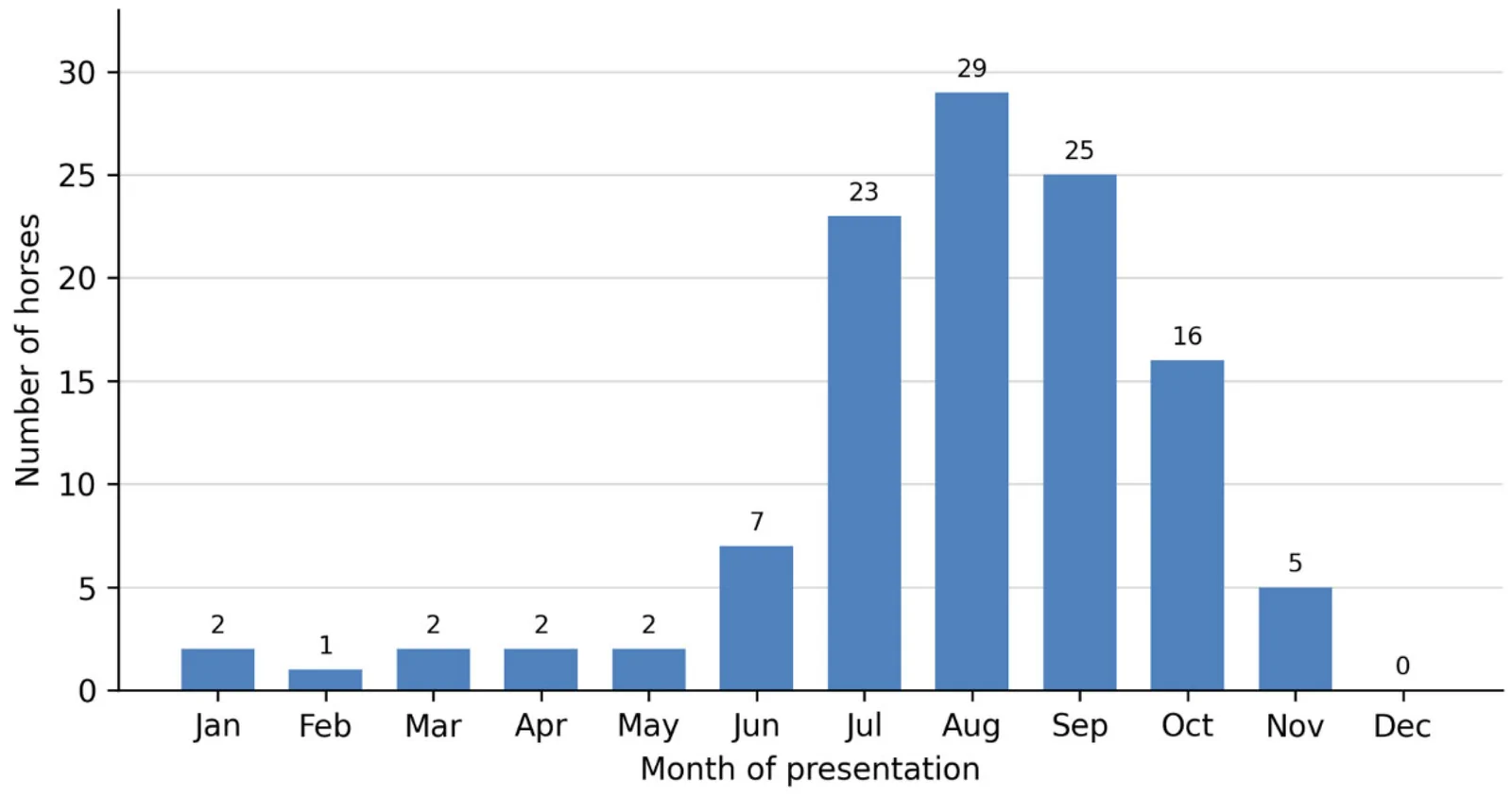
Month of presentation for 114 horses with eosinophilic keratoconjunctivitis between 2012 and 2020.

**Figure 4:**
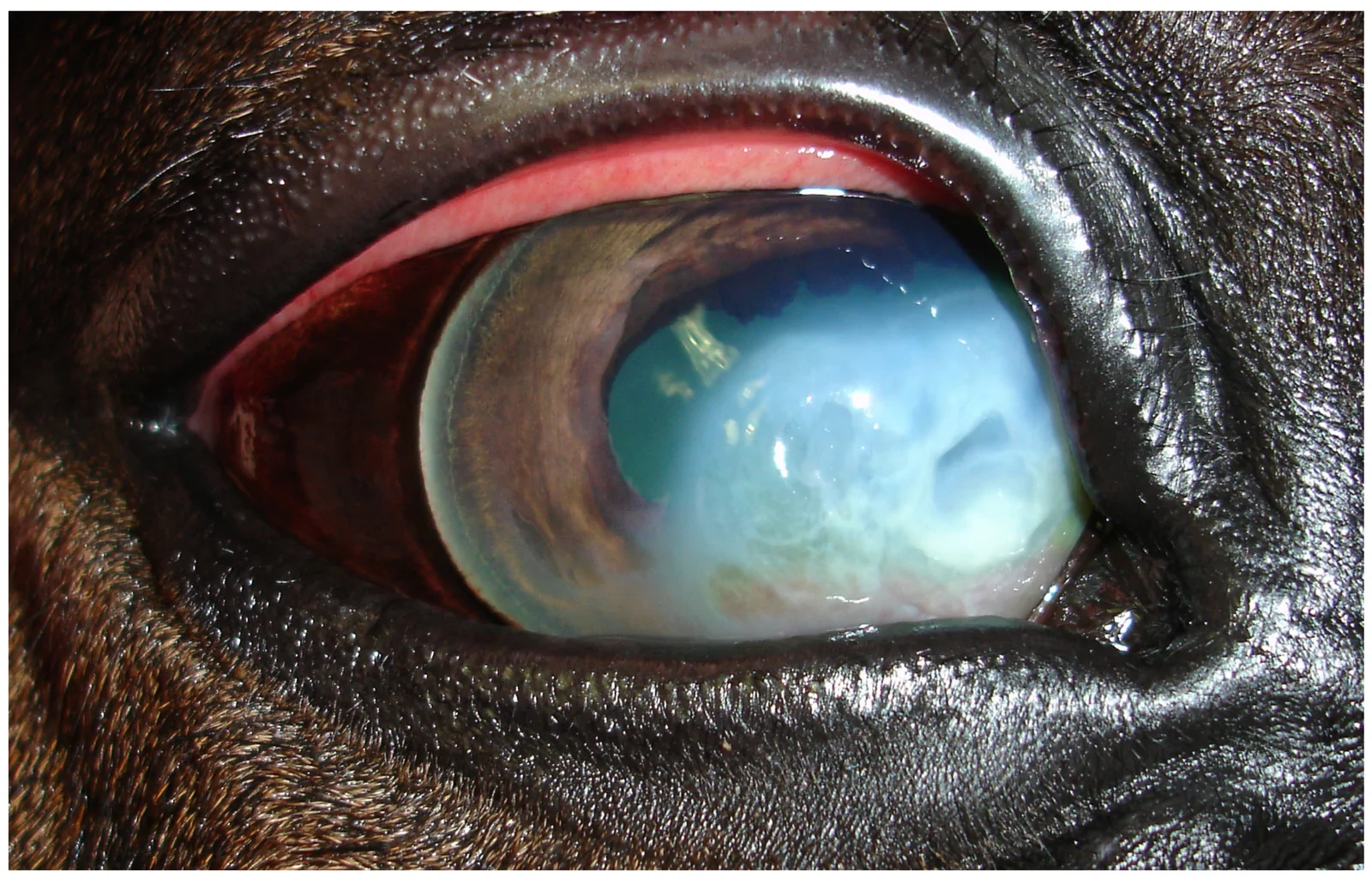
Ulcerative eosinophilic keratoconjunctivitis. A broad corneal epithelial defect involving the central and nasal cornea is accompanied by marked stromal edema.

**Figure 5:**
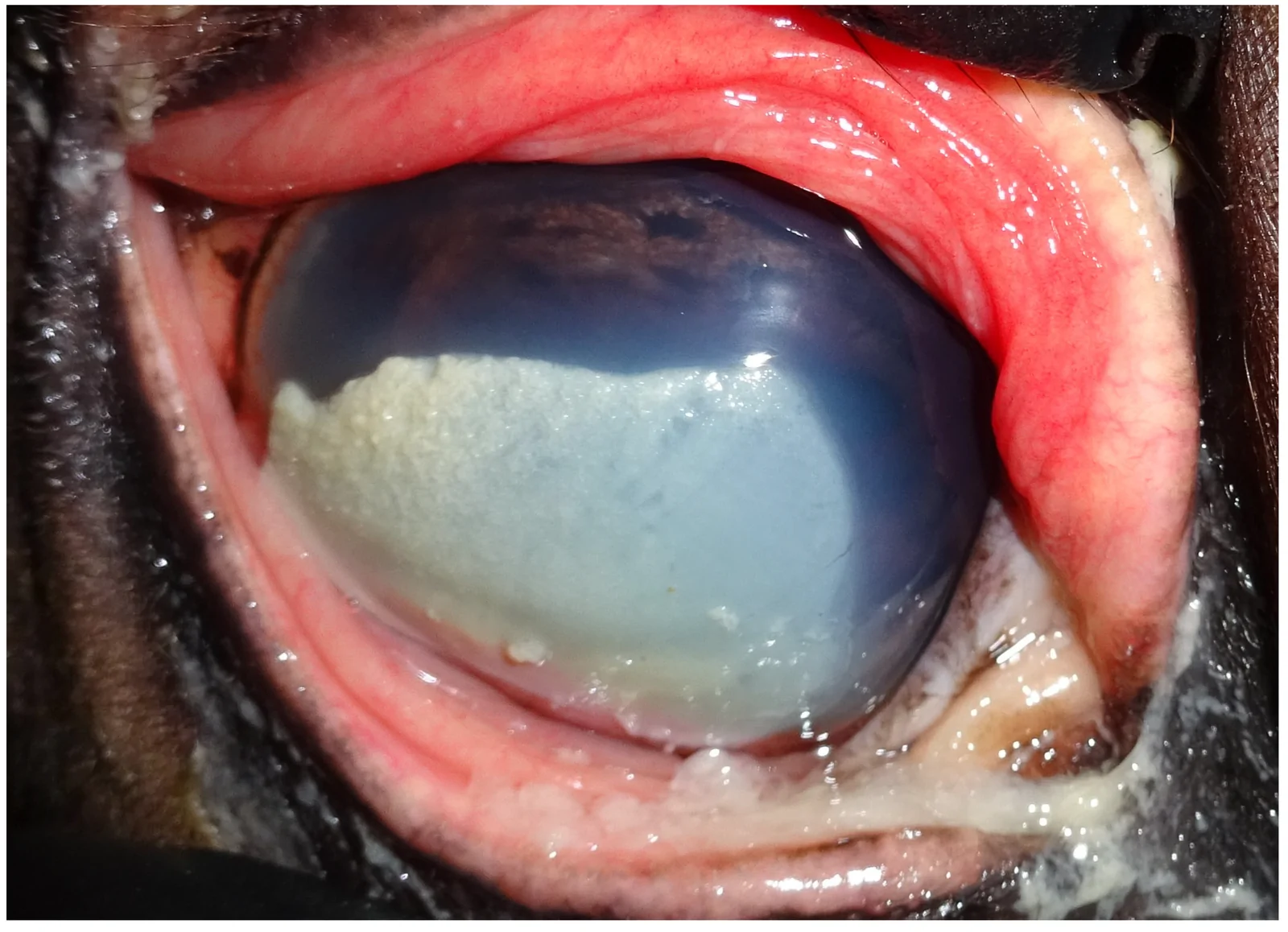
Pseudomembranous/diphtheroid eosinophilic keratoconjunctivitis. A broad, adherent white pseudomembranous plaque covers the central and ventral cornea, with diffuse corneal edema and conjunctival hyperemia.

**Figure 6:**
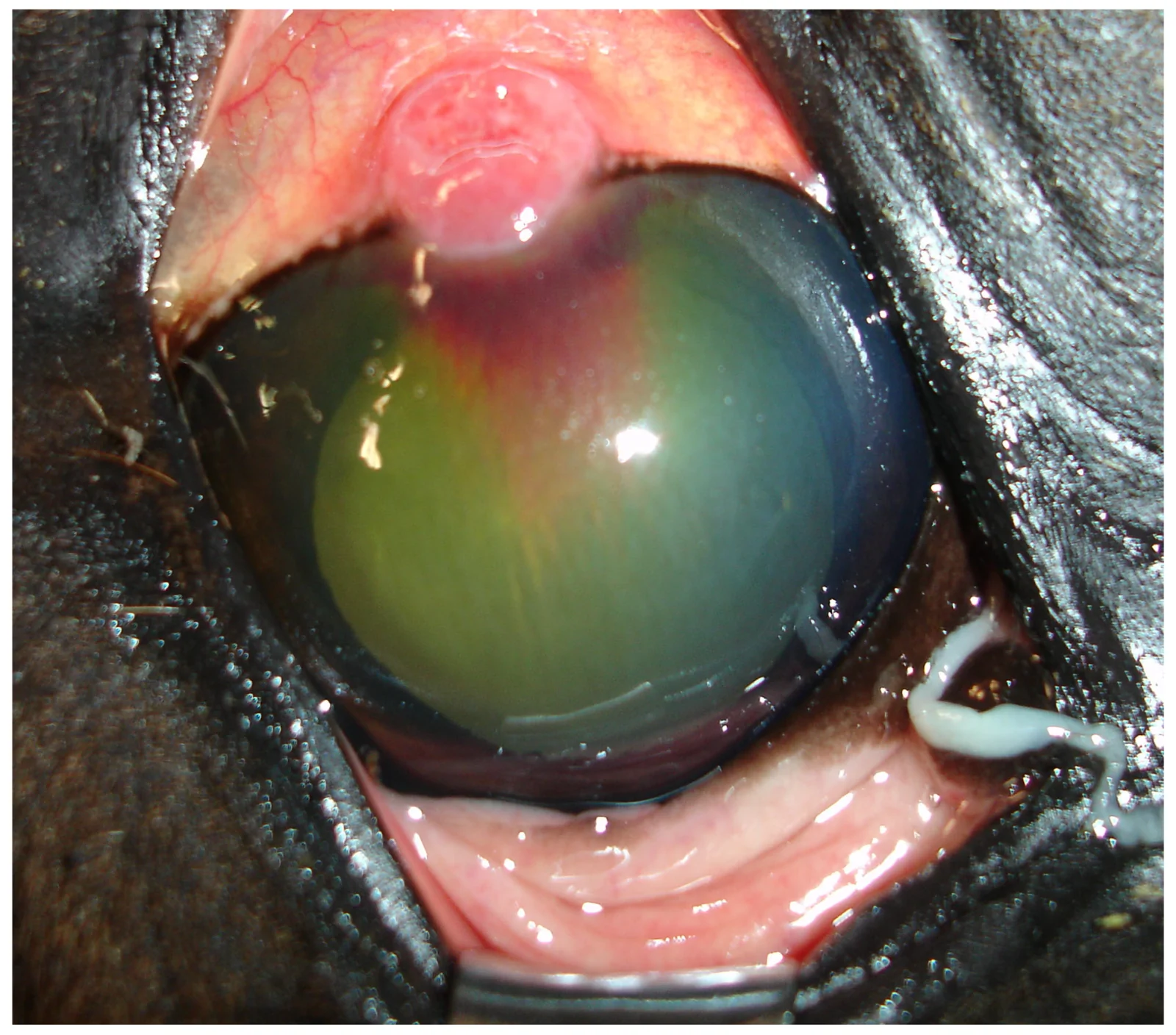
Granulomatous eosinophilic keratoconjunctivitis. A raised pink proliferative lesion is present at the dorsal limbus, with adjacent vascularization and corneal edema.

**Figure 7:**
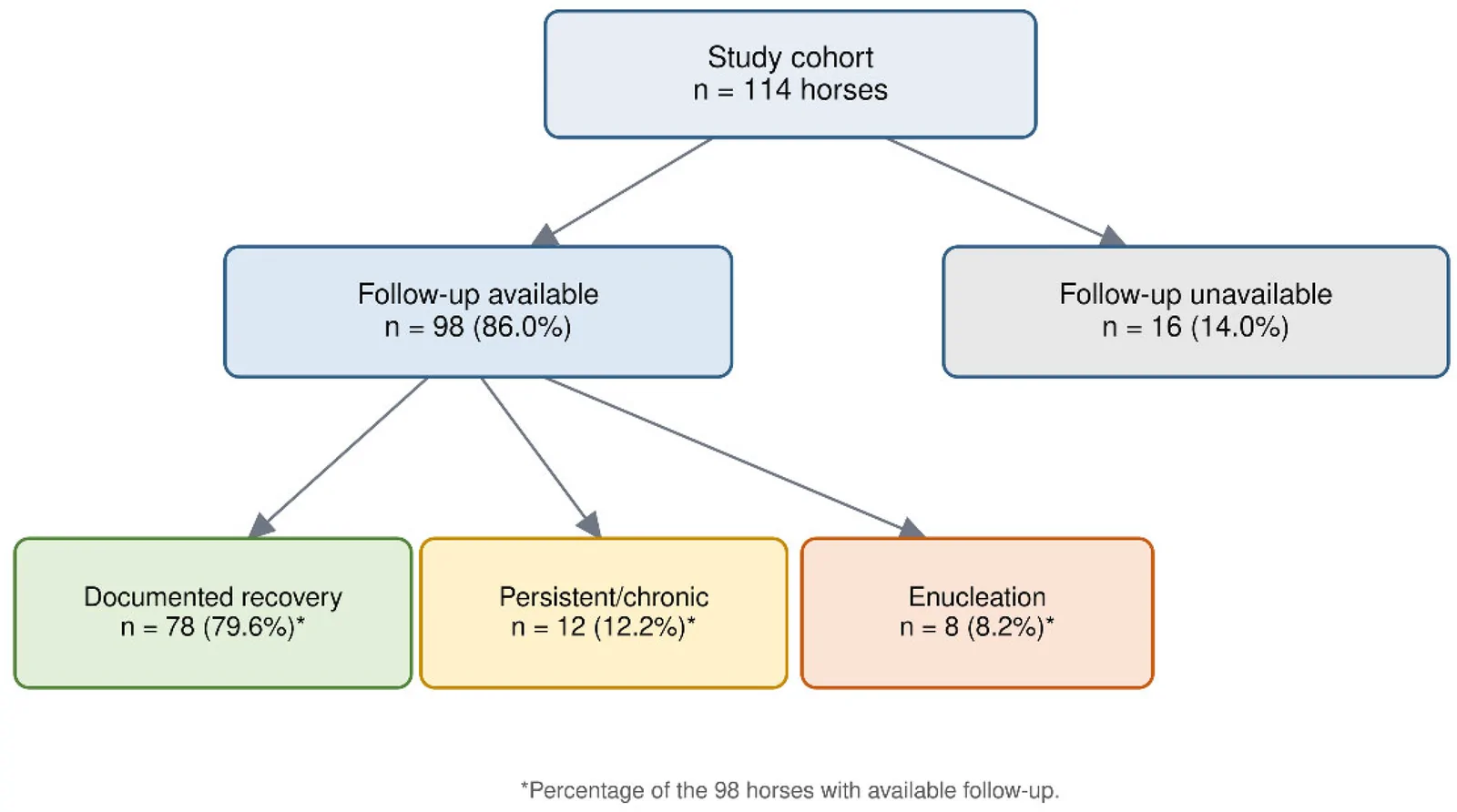
Follow-up availability and documented outcomes in 114 horses with eosinophilic keratoconjunctivitis.

**Table 1:** Summary of the study population, clinical findings, management, and documented outcomes.

Characteristic	*n* or Summary	%/Denominator
Horses/affected eyes	114/163	-
Age, years	11.6 ± 5.1; range 1–22	Mean ± SD
Mares	52	45.6% of horses
Geldings	53	46.5% of horses
Stallions	9	7.9% of horses
Warmbloods	71	62.3% of horses
Unilateral/bilateral disease	65/49	57.0%/43.0% of horses
Cornea involved alone or with another structure	139	85.3% of eyes
Conjunctiva alone	24	14.7% of eyes
Ulcerative/pseudomembranous/ granulomatous corneal lesion	54/29/23	33.1%/17.8%/14.1% of eyes
Mixed corneal lesion	33	20.2% of eyes
Medical treatment alone	24	21.1% of horses
Additional surgical procedure	90	78.9% of horses
Follow-up available/unavailable	98/16	86.0%/14.0% of horses
Recovered/persistent-chronic/enucleated	78/12/8	79.6%/12.2%/8.2% of 98 with follow-up
Recovery time among recovered horses, days	Median 30; IQR 21–60; range 5–330	*n* = 78
Documented recurrence at study hospital	2	2.0% of 98 with follow-up

**Table 2:** Exploratory treatment- and lesion-specific outcomes and recovery times.

Group	Total *n*	Recovered *n*/*N* (%)	Chronic *n*/*N* (%)	Enucleated *n*/*N* (%)	Unknown *n*	Recovery Time Median (IQR), Days
Medical only (horses)	24	22/22 (100)	0/22 (0)	0/22 (0)	2	20.5 (14–30)
Medical plus surgery (horses)	90	56/76 (73.7)	12/76 (15.8)	8/76 (10.5)	14	45 (30–60)
Ulcerative (eyes)	54	47/51 (92.2)	3/51 (5.9)	1/51 (2.0)	3	30 (27.5–60)
Pseudomembranous (eyes)	29	16/26 (61.5)	5/26 (19.2)	5/26 (19.2)	3	60 (30–120)
Granulomatous (eyes)	23	16/18 (88.9)	2/18 (11.1)	0/18 (0)	5	30 (30–45)
Mixed (eyes)	33	21/30 (70.0)	7/30 (23.3)	2/30 (6.7)	3	45 (30–60)

*N* is the number with a known outcome in each row; outcome percentages use *N* as the denominator. Recovery time is summarized only for recovered horses or eyes. Mixed lesions include ulcerative-pseudomembranous, ulcerative-granulomatous, and granulomatous-pseudomembranous forms. Treatment is summarized at horse level and lesion morphology at eye level.

## Data Availability

The original contributions presented in this study are included in the article. Further inquiries can be directed to the corresponding author.

## Abbreviations

The following abbreviations are used in this manuscript:

EKC	eosinophilic keratoconjunctivitis
IQR	interquartile range
SD	standard deviation
